# The relationship between learning burnout, professional commitment, and psychological capital in undergraduate clinical medical students

**DOI:** 10.1097/MD.0000000000035207

**Published:** 2023-09-15

**Authors:** Shaojie Yu, Wei Li, Huizu Yu, Xuehong Ju, Chunguang Ling

**Affiliations:** a School of Psychology, Weifang Medical University, Weifang, Shandong, P.R. China; b Graduate Department, Weifang Medical University, Weifang, Shandong, P.R. China; c Shouguang Hospital of Traditional Chinese Medicine, Shouguang, Shandong, P.R. China.

**Keywords:** clinical medicine, learning burnout, professional commitment, psychological capital, undergraduates

## Abstract

This study examines the current status of and relationship between learning burnout, professional commitment, and psychological capital in undergraduate clinical medical students. A total of 249 undergraduate students were randomly selected from a 5-year clinical medical program at a medical university in Shandong, China. The study employed the Learning Burnout Scale, Professional Commitment Scale, Psychological Capital Scale to survey the sample, and performed structural equation modeling and multiple regression to test the proposed research model using SPSS 19.0. Significant differences in learning burnout were found among students in terms of their gender, years in the medical program, and student leadership (t = 1.811, F = 22.091, t = −2.295; *P* < .01). There were also significant differences in their professional commitment according to their gender, years in the program, and student leadership (t = −2.711, F = 4.275, t = 3.389; *P* < .01). Psychological capital differed significantly based on gender, years in the program, and student leadership (t = 8.709, F = 6.182, *P* < .01, t = 2.086, *P* < .05). Learning burnout showed significant negative correlations with professional commitment and psychological capital (r = −0.311, r = −0.291; *P* < .01). The psychological capital and professional commitment of undergraduate students of clinical medicine serve as predictors of learning burnout. Psychological capital partially mediates the relationship between professional commitment and learning burnout.

## 1. Introduction

Learning burnout refers to a compounded array of academic psychological issues that students face, such as a loss of interest and enthusiasm in studying and a lack of motivation. Observing the learning psychology of university students in China, Griffith defined learning burnout as a scenario in which students become annoyed, develop physical and mental fatigue, and have a negative attitude toward learning activities owing to a lack of motivation or interest.^[1]^ Learning burnout among university students manifests in a series of learning problems, such as obsessive internet surfing and mobile gaming, skipping classes, arriving late, leaving early, and academic dishonesty. These problems profoundly affect students’ academic performance, psychological capacity, and mental well-being, which can lead them to experience a state of confusion and hopelessness about university life.^[2]^

Professional commitment encompasses the positive attitudes and behaviors expressed by students who identify with their chosen field of study and are prepared to exert corresponding effort; it serves as a crucial psychological basis for a student learning initiative and enthusiasm.^[3]^ Research has found a significant negative correlation between all dimensions of professional commitment, as well as overall professional commitment scores, and learning burnout, making professional commitment a significant predictor of learning burnout.^[4]^ Increasing students’ recognition of and emotional connection with their major can, to a certain extent, mitigate learning burnout.^[5]^

Psychological capital is a key variable of positive psychology and refers to a positive psychological state exhibited by individuals during their growth and development. It primarily comprises self-efficacy, hope, optimism, and resilience, and forms an integral component of an individual mental health.^[6]^ Studies exploring the relationship between psychological capital and university students’ learning psychology have observed that it has a significant positive correlation with academic performance: psychological capital indirectly affects academic performance via the mediation of learning strategies^[7]^ and is significantly negatively correlated with learning burnout, thus serving as a crucial predictor of learning burnout.^[8]^

This study examines the current status of learning burnout, professional commitment, and psychological capital among undergraduate clinical medical students, and the interrelation between these factors. The study hypothesizes that professional commitment and psychological capital share a negative correlation with learning burnout, with psychological capital mediating the relationship between professional commitment and learning burnout. Professional commitment can effectively predict the clinical behavior of medical students. Medical students must form excellent professional commitments before internships, which are beneficial for effectively improving clinical performance during the internship process. By exploring the internal mechanism among these 3 variables, our study offers constructive recommendations for improving clinical work among undergraduate clinical medical students.

## 2. Participants and methods

### 2.1. Participants

Five undergraduate students majoring in clinical medicine at a medical university in Shandong were selected as research participants from March 10 to March 22, 2023. We selected 1 class each from grades 1 to 5, and only students with an odd-numbered student ID were chosen to answer the questionnaire. In total, 260 questionnaires were distributed online using the online tool Questionnaire Star. A total of 260 questionnaires were returned, and invalid questionnaires (missed and regular answers) were manually excluded, resulting in 249 valid questionnaires with an effective response rate of 95.38%. The sample size calculation formula (N = 400 Q/P, N = 400(1–68.4%)/68.4%, and N = 185) showed that the sample size of this study was sufficient. The participants comprised 135 men (54.2%) and 114 women (45.8%). Their distribution across the different years in the program was as follows: 55 were first-year students (22.92%); 39 were second-year students (15.66%); 47 were third-year students (18.88%); 58 were fourth-year students (23.29%); and 50 were fifth-year students (20.08%). In addition, 85 respondents held student leadership roles (34.14%), while the remaining 164 students did not (65.86%). All participants signed a consent form. After consulting with the Scientific Research Ethics Committee, we found that our study does not require a review from an ethics board.

### 2.2. Methods

#### 2.2.1. Learning burnout scale.

We adopted the Undergraduate Learning Burnout Scale developed by Lian et al for this study. The scale comprises 25 items divided into 3 dimensions: dejection, improper behavior, and reduced personal accomplishment. A 5-point Likert scoring method was applied, with higher scores indicating a higher degree of learning burnout.^[9]^ The Cronbach alpha coefficients for the subscales of the 3 dimensions were 0.914, 0.779, and 0.704, respectively.

#### 2.2.2. Professional commitment scale.

We also employed the Undergraduate Professional Commitment Scale developed by Lian et al, which consists of 27 items divided across 4 categories: affective commitment, ideal commitment, normative commitment, and continuance commitment. A similar 5-point Likert scoring method was applied, with higher scores indicating a higher degree of professional commitment.^[10]^ The Cronbach alpha coefficients for the 4 subscales were 0.873, 0.891, 0.774, and 0.781, respectively.

#### 2.2.3. Psychological capital scale.

We used Zhang et al’s Positive Psycap Questionnaire, which includes 26 items across 4 dimensions: self-efficacy, hope, resilience, and optimism. A 7-point Likert scale was used, with participants rating each item from 1 (completely disagree) to 7 (completely agree).^[11]^ The internal consistency reliabilities of the 4 dimensions and the total score were 0.846, 0.768, 0.837, 0.742, and 0.910, respectively.

### 2.3. Statistical analysis

SPSS25.0 was used to perform a statistical correlation analysis (learning burnout/ professional commitment/psychological capital) and regression analysis (step 1: learning burnout to professional commitment, step 2: professional commitment to professional commitment, and step 3: learning burnout to professional commitment and psychological capital) on the data.

## 3. Results

### 3.1. Differences in learning burnout, professional commitment, and psychological capital according to demographic variables

There were significant differences in the undergraduate clinical medical students’ levels of learning burnout, professional commitment, and psychological capital according to their gender, years in the program, and student leadership, as shown in Table 1.

**Table 1:** Differences in demographic variables(*X ± S*).

Projects	Learning burnout	Professional commitment	Psychological capital
Male	57.62 ± 12.028	94.78 ± 16.313	118.19 ± 17.993
Female	54.93 ± 11.398	100.04 ± 14.286	101.73 ± 12.941
t	1.811**	−2.711**	8.709**
Grade 1	48.16 ± 11.595	101.29 ± 16.320	118.96 ± 20.780
Grade 2	51.90 ± 6.847	100.21 ± 14.198	112.23 ± 12.345
Grade 3	56.98 ± 11.151	97.46 ± 17.498	109.40 ± 18.241
Grade 4	58.18 ± 11.387	97.71 ± 14.576	102.81 ± 14.545
Grade 5	65.19 ± 8.835	90.43 ± 13.014	108.74 ± 16.047
F	22.091**	4.275**	6.182**
Student cadres	55.16 ± 11.616	99.55 ± 15.663	114.67 ± 17.023
No student cadres	58.75 ± 11.853	92.62 ± 14.540	108.43 ± 17.580
t	−2.295**	3.389**	2.686*

* *P* < .05.

** *P* < .01.

### 3.2. Correlation analysis of learning burnout, professional commitment, and psychological capital

As shown in Table 2, the psychological capital and professional commitment of the undergraduate clinical medical students were significantly positively correlated. Both psychological capital and professional commitment had a significant negative correlation with learning burnout.

**Table 2:** Pearson correlation(*r*).

Projects	1	2	3
Learning burnout	1		
Psychological capitalt	−.291**	1	
Professional commitmen	−.311**	.153*	1

* *P* < .05.

** *P* < .01.

### 3.3. Regression analysis of learning burnout, professional commitment, and psychological capital

We performed a regression analysis with learning burnout as the dependent variable, and professional commitment and psychological capital as predictive variables. The results indicated that both psychological capital and professional commitment predicted learning burnout (see Table 3).

**Table 3:** Regressive analysis.

Dependent variable	Predictive variables	R	R^2^	∆R^2^	F	B	t
Learning burnout	Equation model	0.397	0.158	0.151	23.028	94.929	16.579**
	psychological capital					−0.167	−4.214**
	professional commitment					−0.206	−4.612**

* *P* < .05.

** *P* < .01.

### 3.4. Mediating role of psychological capital in the relationship between professional commitment and learning burnout

The analysis results presented in Table 4 show significant correlations between each pair of variables among psychological capital, professional commitment, and learning burnout. These results satisfy the conditions for analyses of mediating effects as proposed by Du.^[12]^ We conducted a regression analysis to assess the influence of professional commitment (X) on learning burnout (Y), the effect of professional commitment (X) on psychological capital (M), and the combined impact of professional commitment (X) and psychological capital (M) on learning burnout (Y) (see Table 4). The results of the tests (the first 2 and the last t-tests) were all significant, confirming the mediating role of psychological capital in the relationship between professional commitment and learning burnout. The predictive role of professional commitment on learning burnout remained significant when psychological capital was added as the mediator variable. These results suggest that psychological capital partially mediates the relationship between professional commitment and learning burnout.

**Table 4:** Regressive analysis of X, Y, and M.

Step		B	Beta	t
Step 1	Y-X	−0.235	−0.311	−5.148**
Step 2	M-X	0.173	0.153	2.435*
Step 3	Y-X and M	−0.206	−0.273	−4.612**
		−0.167	−0.250	−4.214**

* *P* < .05.

** *P* < .01.

## 4. Discussion

### 4.1. Overview of learning burnout, professional commitment, and psychological capital

In terms of the differences in the learning burnout reported by male and female undergraduate clinical medical students, male students had significantly higher overall scores. Their experience of learning burnout was more intense than that of their female counterparts, which is consistent with the findings of Wu et al.^[13]^ The questionnaire responses revealed that male students were more susceptible to distractions such as computers and online games after entering university, and had poor self-discipline. This lack of self-discipline manifests in behaviors such as arriving late for classes and leaving early, a lack of concentration, falling asleep, and habitual phone use during class, leading to a waning interest and motivation to study.

There were also differences in the learning burnout of undergraduate clinical medical students across different academic years, with burnout escalating as students progressed through the program. According to the questionnaire responses, the difference in burnout across different academic years can be attributed mainly to the initial transition phase from high school to university. During the first year of an undergraduate program, students are typically eager to engage with the unfamiliar content in the university curriculum, and the institution often provides study guidance for incoming students, both of which are factors that can mitigate learning burnout. However, as students advance through the program, the increasing complexity and volume of medical coursework, leading to a stronger sense of academic failure and, consequently, feelings of helplessness. In turn, these feelings result in declining interest, a loss of motivation, and a gradual increase in learning burnout. Students who held leadership positions had a significantly lower levels of learning burnout than their peers, suggesting that being in such positions can sustain professional passion, continually stimulate learning motivation, and encourage diligent, hard-working learning behavior.

In terms of the gender differences in professional commitment among the undergraduate clinical medical students, the overall professional commitment score of male students was significantly lower than that of female students. This result indicates that the level of passion for and identification with the profession among male medical students was markedly lower than among female students. Professional commitment among undergraduate clinical medical students also varied by academic year: the data suggested that students’ professional commitment gradually decreased as they progressed through their program. This decline can be primarily attributed to the increasing academic load, acclimation to the professional field, and subsequent decrease in passion and identification with the profession. Students who took leadership roles exhibited significantly higher professional commitment than their peers, suggesting that they had a greater passion for and identification with their profession.

The male undergraduate clinical medical students had significantly higher scores for psychological capital than the female students, a finding consistent with the work of Doci.^[14]^ The scores for students’ psychological capital were the highest during the first year, followed by the second, third, fifth, and fourth years. These changes in psychological capital align with patterns observed in university students from other majors, which demonstrates that psychological capital decreases as student progress through their programs.^[15]^ The psychological capital of fourth-year students is the lowest since this is the year that represents the transition period from theoretical studies to clinical practice and internships. The alterations in the learning environment and content during this period can have a tangible impact on students’ psychological capital.

### 4.2. Correlation analysis of learning burnout, professional commitment, and psychological capital

Learning burnout in the undergraduate clinical medical students was significantly negatively correlated with psychological capital, which is consistent with the research findings of Barratt et al^[16]^ Additionally, the students’ learning burnout was significantly negatively correlated with professional commitment, supporting the results found by Mei et al^[4]^ The higher the students’ level of professional commitment, the lower the degree of learning burnout they experienced. Similarly, a higher level of psychological capital resulted in a lower level of learning burnout. Based on these findings, we recommend that academic institutions implement systematic professional education from the start of the freshman year and consistently adapt this style to the distinct learning needs of each academic year. In addition, students’ levels of psychological capital can be improved through the provision of systematic mental health education and support.

### 4.3. Regression analysis of the effects of professional commitment and psychological capital on learning burnout

The linear regression analysis revealed that both professional commitment and psychological capital were crucial independent variables affecting learning burnout among the undergraduate clinical medical students, with professional commitment exerting a stronger impact. Therefore, medical universities should intensify the provision of systematic, scientifically-grounded professional education. Elucidating the characteristics, advantages, and development prospects of the medical profession in-depth will allow students to gain a scientific understanding of their profession, thereby stimulating their enthusiasm for and attachment to medicine. This approach can stabilize and enhance students’ professional commitment, which can significantly reduce the learning burnout they experience; communication with patients and medical staff reduce clinical disputes. Students who have significant professional commitment, can establish correct professional values and identity, and enhance the cultivation of clinical communication skills.

### 4.4. Partial mediating role of psychological capital in the relationship between professional commitment and learning burnout

Having established a significant negative correlation between professional commitment and learning burnout, we incorporated psychological capital as a mediating variable for the regression analysis to decipher the internal mechanism linking professional commitment and learning burnout. The regression analysis revealed that psychological capital plays a partial mediating role between professional commitment and learning burnout. Professional commitment can directly influence learning burnout and can also have an indirect effect on learning burnout through psychological capital. Therefore, it is crucial to promote mental health knowledge, offer mental health seminars and group counseling sessions, and focus on the development and cultivation of psychological capital among undergraduate clinical medical students. Leveraging this adaptable, trainable, and positive psychological asset is an important approach to indirectly reducing learning burnout. In clinical work, students with high psychological capital experience less emotional fatigue and more personal sense of achievement. They don’t feel clinical fatigue, thereby enhancing their sense of professional belonging and obligation through hope, optimism, resilience, and happiness.

### 4.5. Strengths and limitations of the research

First, this study is the first to analyze the correlation between learning burnout, psychological capital, and professional commitment. Second, this study conducted a regression analysis using learning variables as the dependent variable, and psychological capital and professional commitment as predictive variables. However, the small sample size could be considered as a limitation of this study. In future research, we will increase the sample size and improve the representativeness of the research sample to provide more references for the growth and development of undergraduate students majoring in clinical medicine.

## 5. Conclusion

This study demonstrates that clinical psychological capital can directly positively affect the professional commitment of undergraduate students majoring in clinical medicine, psychological capital can negatively affect learning burnout, and professional commitment is negatively correlated with learning burnout. Psychological capital plays an important mediating role between professional commitment and learning burnout. Therefore, medical universities and hospitals offering internships should take on practical measures, such as rationalizing internship rotations, improving the clinical environment, and upgrading the level of supervising teachers to enhance students’ positive psychological experience. They should also strengthen education for students so that they can demonstrate a positive psychological state to reduce learning burnout and strengthen professional identity.

## Author contributions

**Conceptualization:** Chunguang Ling.

**Data curation:** Wei Li, Huizu Yu.

**Formal analysis:** Shaojie Yu, Chunguang Ling.

**Funding acquisition:** Xuehong Ju.

**Investigation:** Shaojie Yu.

**Methodology:** Shaojie Yu, Chunguang Ling.

**Project administration:** Huizu Yu.

**Resources:** Huizu Yu.

**Software:** Wei Li.

**Writing – original draft:** Shaojie Yu, Chunguang Ling.

**Writing – review & editing:** Shaojie Yu, Chunguang Ling.
